# Inference About Absence as a Window Into the Mental Self-Model

**DOI:** 10.1162/opmi_a_00206

**Published:** 2025-04-22

**Authors:** Matan Mazor

**Affiliations:** Department of Experimental Psychology and All Souls College, University of Oxford, Oxford, UK

**Keywords:** self-model, absence, metacognition

## Abstract

To represent something as absent, one must know that they would know if it were present. This form of counterfactual reasoning critically relies on a *mental self-model*: a simplified schema of one’s own cognition, which specifies expected perceptual and cognitive states under different world states and affords better monitoring and control over cognitive resources. Here I propose to use inference about absence as a unique window into the structure and function of the mental self-model. I draw on findings from low-level perception, visual search, and long-term memory, in support of the idea that self-knowledge is a computational bottleneck for efficient inference about absence, and show that alternative “direct perception” and “heuristic” accounts either fail to account for empirical data, or implicitly assume a self-model. I end with a vision for an empirical science of self-modelling, where inference about absence provides a cross-cutting framework for probing key features of the mental self-model that are not accessible for introspection.

## INTRODUCTION

You are in the grocery shop. On your grocery list are one carton of oat milk and one durian: a tropical fruit known for its intense fragrance. You search through the shelves and find your favourite oat milk. You place the carton in your basket and move on to the fruit aisle. You visually scan the fruit boxes, but you already have a strong feeling that you will not find durians in this store. You would have already smelled the durians if they were anywhere around you. But then again, maybe something is wrong with your sense of smell? You grab a mandarin and sniff it. Your sense of smell is intact. You can be confident that there are no durians around.

## INFERENCE ABOUT ABSENCE

Finding the oat milk carton was straightforward. As soon as you identified it you were convinced in its presence, no reflection or deliberation required. In contrast, concluding that no durians were present took you longer and involved more complex cognitive processes. You had to rely on the absence of smell or sight of the fruit to reach a conclusion. In philosophical writings, this is known as Argument from Ignorance (*Argumentum ad ignorantiam*): the fallacy of accepting a statement as true only because it hasn’t been disproved (Locke, 1948). Although logically unsound, *Argumentum ad ignorantiam* is widely applied by humans in different situations and contexts, and specifically in inference about absence. Positive evidence is rarely available to support inference about absence, and so it is often made on the basis of a failure to find evidence for presence.

Basing inference on the absence of evidence is not always fallacious, and can sometimes be rational from a Bayesian standpoint (Oaksford & Hahn, 2004). For this to be the case, the individual must know the sensitivity and specificity of the perceptual or cognitive system at hand. For example, in order for the inference “I don’t smell a durian, therefore there are no durians in this store” to be logically sound, I need to know that the probability of me not smelling a durian is very low when nearby, and so is the probability of me imagining the smell of a durian when it is not there. In other words, in order to make valid inferences about absences I need to know things about myself and my cognitive processes. In the above example, this is evident in that my certainty in the absence of a durian increased after smelling the mandarin. Critically, smelling the mandarin did not provide me with any additional information about the layout of the shop or the seasonal availability of tropical fruit, but about my own perceptual system.

This example of inference about absence is exceptional in that I am able to justify my reasoning. If my friend later asks me why I concluded that no durians were in the store, I can convince them by explaining how I normally smell durians from a distance, that I was able to smell the mandarin, and that I concluded that I would have detected a durian if it were present. But explicitly representing a derivation chain from assumptions to conclusions is the exception, not the rule. I know with confidence that there is no glass of water on my desk right now. If my friend were to ask me how I concluded that there was no glass of water on my desk, I would probably answer that I could see that it was not there. But this does not mean that I perceived its absence. It means that I did not perceive its presence, and that I believe I *would have* perceived it if it were there. The first part is a fact about my perception, but the second part is a belief about my perceptions in a counterfactual state of affairs in which a glass of water were present on my desk (Mazor et al., 2024; Seth, 2014). This belief builds on my knowledge of water glasses, but more relevant to us here, on a *mental self-model*: a structured representation of one’s own cognition, perception and attention that allows agents to predict their sensations, thoughts, and actions under different world states. Such model-based predictions can be made about hypothetical states (“*what would I sense if this happens?*”), future states (“*what will I sense in a minute from now?*”) or, as in the absent water glass example, counterfactual ones (“*what would I have sensed if this were the case?*”).

Self-models are a subcategory of internal models, broadly defined here as structured representations of target systems. Internal models are distinct from lists of facts in that they are generative: they can be used to generate predictions about the behaviour of the system under infinitely many settings, even ones that were never encountered before. For example, an internal model of the physical world allows humans to predict the trajectories of falling balls and the stability of brick towers (Battaglia et al., 2013; Zago et al., 2009). A second property of internal models is that they often (but not always) comprise non-declarative knowledge, that is, they can guide behaviour and decision-making even if their content cannot be reported. For example, forward-models in motor control (Miall & Wolpert, 1996; Wolpert et al., 1998) are simplified internal representations of one’s motor system and body that can be used to translate motor commands to expected sensory input (for example, an expectation to hear a certain voice when allowing air through the vocal cords). The rich knowledge that is specified in the forward model is not necessarily available to report, but guides our behaviour in a phenomenally-transparent manner. Finally, internal models are often simplified: to be useful, models need to balance between simplicity and exactitude, often ignoring details and focusing on general laws.

A self-model is such a generative, non-verbal, and simplified knowledge structure that describes the perceptual and cognitive system of the agent itself. Self-models have been suggested to play an important role in attention control (Wilterson et al., 2020), theory of mind (Graziano, 2019), and subjectivity more generally (Metzinger, 2003). It is likely that parts of the model are not available to introspection at all (in contrast to explicit beliefs and narratives we may hold about ourselves), but affect our behaviour in interesting ways nonetheless (Flavell, 1979). Here I argue that this necessary role for a self-model in inference about absence makes such inferences a promising tool to probe people’s self-models.

The following section introduces a formulation of this self-knowledge account, based in formal semantics and Bayesian theories of cognition, and exemplifies how different patterns of results can be interpreted in light of this formulation. This formulation is then followed by descriptions of several independent lines of experimental work that all share a role for self-knowledge in inference about absence. Finally, I present a vision for how future work can utilize these mechanisms to learn about the structure of this knowledge and about its acquisition over the course of development.

### Inference About Absence and Epistemic Closure

This paper is not the first to point out the intimate link between inference about absence and self-knowledge. In *default-reasoning logic* (Reiter, 1980), a failure to provide a proof for a statement is transformed into a proof for the negation of the statement using the *closed world assumption*: the assumption that a proof would have been found if it were available. Similarly, linguist Benoît de Cornulier refers to *epistemic closure*: the notion that all there is to be known is in fact known. This is reflected in his two definitions of *knowing whether* (de Cornulier, 1988):

**Symmetrical definition:**

‘John knows whether P’ means that:

1. If P, John knows that P.
2. If not-P, John knows that not-P.

**Dissymmetrical definition:**

‘John knows whether P’ means that:

1. If P, John knows that P.
2. John knows that 1 holds.

The symmetrical definition can be applied when a statement can be supported or negated by evidence. For example, the statement “It is not yet 3 pm” can be supported if the time on one’s phone indicates that it is 2:30 pm, or negated if the time on one’s phone indicates it is 3:30 pm. Therefore, knowing whether it is not yet 3 pm does not rely on self-knowledge. Conversely, statements such as “I have met this person before” can only be supported by positive evidence. In the majority of cases, no evidence is available to support inferences about the absence of objects or memories. This leaves such inferences to be made based on the absence of evidence, in conjunction with beliefs about the availability of counterfactual evidence: a form of belief that critically depends on self-knowledge (“*I don’t recall seeing this person before, and this is not a face that I would forget*”). This is an example of de Cornulier’s dissymmetrical definition: knowing that I would not have forgotten this person’s face is in this case ‘knowing that 1 holds’.

**Figure 1.**
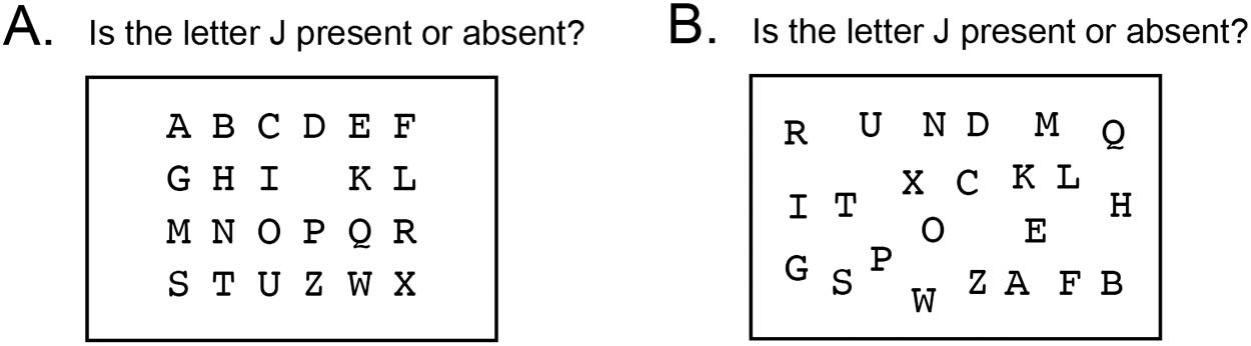
Inferences about absence and the precision of expectations about evidence for presence. A: a precise expectation to find the letter J between I and K makes its absence salient. B: an imprecise expectation makes the same inference harder, and confidence in the inference lower.

A further consequence of the dissymmetrical definition is that the persuasion with which inferences can be made about absence scales with the precision of the expectation regarding evidence for presence. For example, I will be more persuaded in the absence of a durian if I know that, whenever a durian is present, it is placed in a specific section in the store. My level of persuasion in absence will be even higher if I know that durians, when present, are put on a specific shelf. When the prior about the nature of counterfactual evidence for presence is strong enough (for example, when the absent object is part of a familiar or a visually organised pattern), inference about absence can be made quickly and with high confidence. Decisions about absence can therefore be organised along a gradient, ranging from omissions (cases where the prior over evidence for presence is highly precise, see Figure 1A), though relatively constrained inferences about absence (see Figure 1B), and all the way to uncertain and tentative inferences about absence (for an interactive example, see Appendix A).

### Default-Mode Reasoning in Psychological Models of Inference About Absence

In psychological experiments of near-threshold detection, participants are required to decide whether a stimulus (for example a faint grating) was present or absent. Using de Cornulier’s formulation, we can ask which of the two definitions better describes the inferential machinery that is put to work in such tasks. Is it the case that participants perceive positive evidence for the absence of a target (symmetrical definition), or alternatively, do they rely on the metacognitive belief that they would have seen the target if it were present (dissymmetrical definition)?

**Figure 2.**
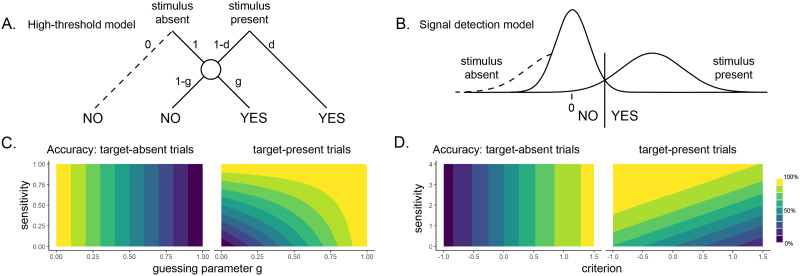
Upper panels: state and strength models of detection, commonly used in visual perception and recognition memory. A. In discrete high-threshold models, the presence of a signal can directly lead to a ‘yes’ response, but the absence of a signal is never sufficient to lead to a ‘no’ response. ‘No’ responses are controlled by the parameter *g*—a ‘guessing parameter’ that determines the probability of responding ‘yes’ in case no stimulus was detected. The dashed line represents the missing direct link from stimulus absence to a ‘no’ response. B. In unequal-variance SDT models, decisions are made based on the position of the sensory sample relative to a decision criterion. Only in some ‘target-present’ trials, but not in ‘target-absent’ trials, the sensory sample falls far away from the decision criterion, giving rise to a presence/absence asymmetry. The dashed line represents the missing long tail of the ‘stimulus absent’ distribution. Lower panels C and D: the effects of model parameters on accuracy in target-absent and target-present trials (hit and correct rejection rates). Accuracy in target-absent trials is affected only by parameters that are under subjects’ metacognitive control.

The *high-threshold model* of visual detection (Blackwell, 1952) formalizes this process in a way that shares conceptual similarity with de Cornulier’s dissymmetrical definition (see Figure 2A). In the model, the probability of detecting a signal is assigned to the variable *d*. This variable scales with stimulus intensity, and thus can be considered a perceptual parameter: it captures variables such as objective stimulus intensity (for example, in units of luminance) and sensory sensitivity (for example, of photoreceptors in the retina, or neurons in the visual cortex). If participants detect the signal, they respond with ‘yes’. Thus, *d* corresponds to the degree to which statement 1 in the dissymmetrical definition is true: “If P [a stimulus is presented] John knows that P”. Critically, in the high-threshold model no similar parameter exists to control the probability of detecting the absence of a signal. In Figure 2A, the presence/absence asymmetry is expressed in the absence of a direct edge from ‘stimulus absent’ to a ‘no’ response. In this model, ‘no’ responses are controlled by the ‘guessing’ parameter *g*. Unlike *d*, the *g* parameter is under participants’ cognitive control, and can be optimally set to maximize accuracy based on beliefs about the probability of a stimulus to appear, the incentive structure, and critically, metacognitive beliefs about the perceptual sensitivity parameter *d*.

The high-threshold model, like other discrete state accounts of perception, has mostly been neglected in light of evidence of graded perception, even for sub-threshold stimuli (e.g., Koenig & Hofer, 2011). Still, continuous and graded models of perception based on Signal Detection Theory (SDT) express the same asymmetrical nature of presence/absence judgments, where clear evidence can be available for presence but less so for absence. In signal detection terms, this is expressed as high between-trial variance in perceptual evidence when a signal is present, but low variance when a signal is absent (see Figure 2B). Here, instead of controlling the parameter *g*, participants control the placement of a decision criterion. Only trials in which perceptual evidence (also termed the decision variable) exceeds this criterion will result in a “target present” response. Optimal positioning of the criterion is dependent on beliefs about the prior probability of target presence, as well as the spread of the signal and noise distributions and the distance between them. Due to the unequal-variance structure, perceptual evidence in trials where a stimulus is present will be on average farther from the decision criterion compared to when no stimulus is present. As a result, similar to the setting of the *g* parameter in the high-threshold model, the exact placement of the SDT decision criterion will have a stronger effect on accuracy when a stimulus is absent, compared to when a stimulus is present.

Common to both frameworks is the reliance on knowledge about one’s own perception (the *d* parameter in the first case, the shape and position of the sensory distributions in the second) for optimally setting a response strategy on trials in which no clear evidence is available for the presence of a signal. Indeed, as can be seen in Figures 2C and 2D, when a target is present detection accuracy is a product of both sensitivity and response strategy, but in the absence of a target accuracy is solely determined by parameters that control response strategy. As a result, these models draw a strong link between participants’ beliefs about their own perception and their behaviour on target-absent trials.

In what follows I show that inferences about the presence or absence of objects and memories exhibit robust behavioural asymmetries. I then link those examples to the core idea, that inference about absence critically relies on access to a self-model. Finally, I demonstrate how this link can be utilized by researchers to investigate participants’ mental (perceptual and cognitive) self-models.

## DETECTION: “I WOULD HAVE NOTICED IT”

We start our exploration of inference about absence in cognition with perhaps the most basic psychophysical task: visual detection. In visual detection, participants report the presence or absence of a target stimulus, commonly presented near perceptual threshold. In such tasks, accuracy alone cannot reveal a difference in processing between decisions about presence and decisions about absence, because task accuracy is a function of both ‘yes’ and ‘no’ responses.

However, when asked to report how confident they are in their decision, subjective confidence reports reveal an asymmetry between judgments about presence and absence. Decisions about target absence are accompanied by lower confidence, even for correctly rejected ‘stimulus absence’ trials (Kanai et al., 2010; Mazor et al., 2020, 2021; Meuwese et al., 2014). Put differently, often participants cannot tell if they missed an existing target, or correctly perceived the absence of a target. A similar pattern is observed for response times: decisions about absence tend to be slower than decisions about presence (Mazor et al., 2020, 2021).

These observations fit well with the asymmetric unequal-variance SDT model described above (Kellij et al., 2021). An unequal-variance setting (whether produced by physiological constraints on neuronal firing rates, or by physical properties of the stimuli themselves) limits the availability of evidence for absence, making inference about absence more challenging. Only in the presence of a target stimulus can participants make a decision without deliberation (without passing in the *A* node in the high-threshold model, or based on a sample very far from the decision criterion in unequal-variance SDT). On these trials, participants can be highly confident in the presence of a target. In unequal-variance SDT models, decisions about target absence are almost never driven by a sample far away from the decision criterion, and so can not be accompanied by similarly high levels of confidence.

In line with a central role for self-monitoring in inference about absence, this presence-absence asymmetry diminishes or reverses when targets are masked from awareness by means of an attentional manipulation (Kanai et al., 2010; Kellij et al., 2021). For example, when an attentional-blink paradigm is used to control stimulus visibility, participants are significantly more confident in their ‘no’ responses when the target stimulus is indeed absent. What is it in attentional manipulations that improves metacognitive insight into judgments about stimulus absence? One compelling possibility is that a blockage of sensory information at the perceptual stage is not accessible to awareness, whereas fluctuations in attention are (Kanai et al., 2010). This monitoring of one’s attention state makes it possible to use premises such as “I would not have missed the target” in rating confidence in absence under attentional, but not under perceptual manipulations of visibility. Put in more formal terms, attentional manipulations increase metacognitive access to the likelihood function going from world-states to perceptual states, thereby allowing trial-to-trial tuning of the decision criterion.

**Figure 3.**
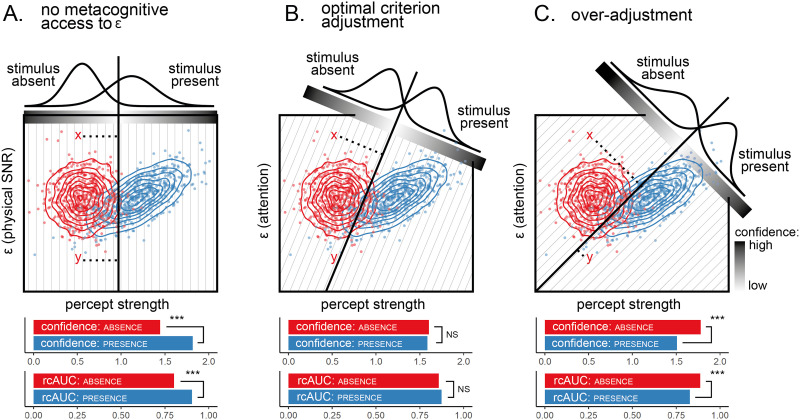
Simulation results from signal detection models of perception under degraded vision (panel A) and attentional load (panels B and C). The decision criterion is shown as a solid straight line. Here, decision evidence is defined as the signed distance from the decision criterion (illustrated for points *x* and *y* with a dashed line). The distributions of decision evidence are shown for the three conditions. Note that the distribution of perceptual evidence (plotted along the *x*-axis) is the same in all panels; differences in decision evidence distributions result solely from variations in the decision criterion’s orientation across panels. Confidence is modeled as the distance from the decision criterion, indicated by a grayscale gradient. A) When stimulus visibility is degraded by added noise (lower SNR), decision evidence is more variable across trials when a stimulus is present. As a result, both confidence and metacognitive sensitivity (measured as the area under the response-conditional ROC curve, rcAUC) are lower for absence decisions. B) When stimulus visibility is degraded by an attentional manipulation (note the change in the *y*-axis label), subjects can adjust their decision criterion based on their current level of attention. As a result, confidence and metacognitive sensitivity are equal for presence and absence decisions. C) If subjects overestimate the effect of attention on percept strength, they will over-adjust their criterion. As a result, confidence and metacognitive sensitivity will be higher, rather than lower, for absence decisions. Note the effects of the different criteria on the classification and confidence of points *x* and *y*, which share the same percept strength but differ in epsilon values. Lower panels: group statistics from 100 identical simulated agents, each completing 100 trials. **p < .001; NS = non-significant.

Figure 3 illustrates this model-based criterion adjustment. In all three panels, the underlying generative model is the same: percept strength is sampled from the normal distribution N(0, 1) on target-absent trials, and from N(3 + *ϵ*, 1) on target-present trials, where *ϵ* is a latent variable that follows a normal distribution *ϵ* ∼ N(0, 1). If subjects do not have access to fluctuations in *ϵ* (as expected when visibility is manipulated by means of factors that are external to the subjects, such as phase scrambling), the decision criterion is independent of *ϵ*, and confidence (measured as the absolute distance of the perceptual sample from the decision criterion) is both higher and more aligned with objective accuracy in decisions about presence (Figure 3A). Having access to the value of *ϵ* (as is the case when visibility is manipulated by degrading attention) allows subjects to adjust their decision criterion by making it more conservative when stronger percepts are expected, rendering confidence judgments similar in decisions about presence and absence (Figure 3B).

Interestingly, in a recent study employing an attentional blink paradigm, confidence ratings were more consistent with a wider distribution of perceptual evidence in target-absent, rather than target-present trials (Kellij et al., 2021). This flipped pattern is expected if subjects over-adjust their decision criterion as a function of *ϵ*, for example due to miscalibrated beliefs about the effects of attention on perception (Figure 3C). Such a change to the decision criterion should not affect perceptual evidence itself (the *x* axis in the upper panels of Figure 3), but it would affect the distribution of decision evidence: the signed distance of perceptual samples from the decision criterion (presented as gaussian distributions in the same panels).

## VISUAL SEARCH: “I WOULD HAVE FOUND IT”

In visual search tasks, participants are presented with an array of stimuli and are asked to report, as quickly and accurately as possible, whether a target stimulus was present or absent in the array. Moving one step up the complexity ladder, the accumulation of information in visual search is not only a function of stimulus strength and sensory precision, but is also affected by the allocation of attention to items in the visual array. As a result, search time varies as a function of the number of distractors, their perceptual similarity to the target and their spatial arrangement, among other factors (for a review, see Wolfe & Horowitz, 2008). These factors affect not only the time taken to report the presence of a target, but also the time taken to report its absence. For example, when searching for an orange target among red and green distractors, the number of distractors has virtually no effect on search time in both target-present and target-absent trials (e.g., D’Zmura, 1991)—a phenomenon known as ‘pop-out’. The bottom-up pop-out of a target can explain the immediate recognition of the presence of a target, irrespective of distractor set size. But this perceptual pop-out cannot, by itself, explain the immediate recognition of target absence, because in target-absent trials there is nothing in the display to pop out.

Computational models of visual search provide different accounts for search termination in target-absent trials. For example, in some versions of the *Guided Search* model, ‘target absent’ judgments are the result of exhausting the search on items that surpassed a learned ‘activation threshold’ (Chun & Wolfe, 1996; Wolfe, 1994). In difficult searches, the activation threshold is set to a low value, thereby requiring the scanning of multiple items before a ‘no’ response can be delivered. In contrast, in easy searches the activation threshold can be set to a high value, reflecting a belief that a target would be highly salient. More recent models include a *quitting unit* that can be chosen with a certain probability (Moran et al., 2013) or a *quitting threshold* parameter that resembles a noisy timer on search duration (Wolfe, 2021). Importantly for our point here, these different parameters all share high similarity with the SDT criterion or the high-threshold *g* parameter, and reflect explicit or implicit beliefs about the subjective salience of a hypothetical target in the array—a form of self-knowledge.

**Figure 4.**
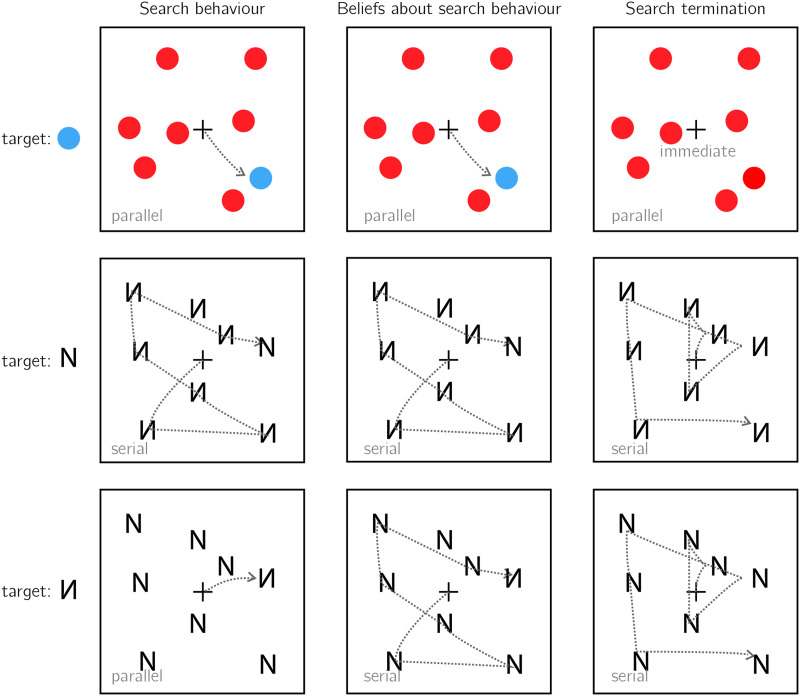
Upper panel: A target marked by a unique colour immediately captures attention (left). This fact is represented in participants’ self-model (middle). As a result, participants can immediately terminate the search when no distractor shares the target’s colour (right). Middle panel: When searching for the letter N among inverted Ns, the target does not immediately capture attention, and a serial deployment of attention is required (left). Participants recognize this (middle). As a result, participants perform an exhaustive serial search before concluding that no target is present (right). Lower panel: When searching for an inverted N among upright Ns, the inverted letter immediately captures attention (left). This fact is not represented in the self-model (middle). As a result, participants perform an unnecessarily exhaustive serial search before concluding that no target is present (right).

Usually, search times in target-present and target-absent trials are highly correlated, such that if participants take longer to find the target in a given display, they will also take longer to conclude that it is absent from it (Wolfe, 1998). This alignment speaks to the accuracy of the mental self-model: participants take longer to conclude that a target is missing when they believe they would take longer to find the target, and these beliefs about hypothetical search times are generally accurate. In the two upper panels of Figure 4 I provide two examples of cases where beliefs about search behaviour perfectly align with actual search behaviour, leading to optimal search termination. However, self-knowledge about attention in visual search is not always accurate. For example, when searching for an unfamiliar letter (for example, an inverted N) among familiar letters (for example, Ns), the unfamiliar letter draws immediate attention without a need for serially attending to each item in the display. Still, participants are slow to infer the absence of an unfamiliar letter, exhibiting a search time pattern consistent with a serial search for ‘target absent’ responses only (Wang et al., 1994). In the context of my proposal here, this can be an indication for a blind spot of the mental self-model, failing to represent the fact that an unfamiliar letter would stand out (see Figure 4, lower panel).

Importantly, collecting explicit metacognitive judgments of expected search times may lead to underestimating the richness and accuracy of the mental self-model. For example, participants have no introspective access to their knowledge about color pop-out (Mazor et al., 2023), while still being able to act on this information when deciding to terminate their search. Here also, inference about absence provides a unique window into the mental self-model that does not depend on introspective access.

## MEMORY: “I WOULD HAVE REMEMBERED IT”

We can infer the absence not only of external objects (such as durians, or visual items on the screen), but also of mental variables such as memories and thoughts. For example, upon being introduced to a new colleague, one can be certain that they have not met this person before. In the memory literature, this is known as *negative recognition*: remembering that something did not happen (Brown et al., 1977). In the lab, this is studied by presenting participants with a list of items, and asking them later to classify different items as ‘old’ (presented in the learning phase) or ‘new’ (not presented in the learning phase). Negative recognition is then defined as subjects’ ability to classify unlearned items as ‘new’.

The role of self-knowledge in negative recognition is exemplified in the *mirror effect*: items that are more likely to be correctly endorsed as ‘old’ are also more likely to be correctly rejected as ‘new’. For example, Brown et al. (1977) found that when asked to memorize a list of names, subjects are more confident in remembering that their own name was on the list, and critically, they are also more confident in correctly remembering when it was *not* on the list. For this effect to manifest, it is not sufficient that subjects’ memory was better for their own name. They also had to know this fact, and to use it in their counterfactual thinking (“*I would have remembered if my name were on the list*”). The mirror effect has also been demonstrated for the name of one’s hometown (Brown et al., 1977), for word frequency (rare words are more likely to be correctly endorsed or rejected with confidence; Brown et al., 1977; Glanzer & Bowles, 1976), word imaginability (Cortese et al., 2010, 2015) and for study time (subjects are more likely to correctly reject items if learned items are presented for longer; Starns et al., 2012; Stretch & Wixted, 1998).

In a clever set of experiments, Strack et al. (2005) established a causal effect of metacognitive beliefs about item memorability on decisions about the absence of memories. In two experiments, participants in one group were led to believe that high-frequency words (words that are used relatively often) are more memorable than low-frequency words, while participants in a second group were led to believe that low-frequency words were more memorable than high-frequency words. This manipulation affected participants’ negative recognition judgments in a later recognition-memory task. Participants who believed that high-frequency words were more memorable were more likely to classify high-frequency words as ‘new’, suggesting that their metacognitive belief informed their inference about the absence of a memory (“*I would have remembered this word*”). Inversely, participants who believed that low frequency words were more memorable showed the opposite pattern.

Just like in the cases of near-threshold detection and visual search, the intuitive metacognitive knowledge behind the mirror effect may not be available for explicit report, at least not in the absence of direct experience with the task itself. In their explicit memorability reports, subjects often have little to no declarative metacognitive knowledge of which items are more likely to be remembered, even under conditions that give rise to a mirror effect. For example, although more frequent words are more likely to be forgotten (and incorrectly classified as old), participants tended to judge them as *more* memorable than infrequent words (Begg et al., 1989; Benjamin, 2003; Greene & Thapar, 1994; Wixted, 1992). Similar to beliefs about perceptual sensitivity in the visual periphery or beliefs about attention in visual search, this may be one example where using inference about absence to probe self-knowledge can reveal more than what can be measured with explicit subjective reports.

## DOES INFERENCE ABOUT ABSENCE REALLY REQUIRE A SELF-MODEL?

This paper focuses on the role of self-modelling in inference about absence. But some readers may feel that this is a stretch: in many occasions, absence can be inferred without any self-model or self-representation at all, based on the direct perception of absence or uniformity, or on learning of task statistics. In the following I describe these two approaches, and show that they do not explain inference about absence in some cases, or implicitly require a self-model in others.

### Direct Perception of Absence

According to some contemporary philosophers, absence need not be inferred because it is directly perceived. For example, philosopher Anna Farennikova explains the perception of absence as a perception of a mismatch between sensory input and expectations of presence: “The phenomenology of absence is the experience of incongruity” (Farennikova, 2013). Farennikova presents the following example of absence perception:

“You’ve been working on your laptop in the cafe for a few hours and have decided to take a break. You step outside, leaving your laptop temporarily unattended on the table. After a few minutes, you walk back inside. Your eyes fall upon the table. The laptop is gone! This experience has striking phenomenology. You do not infer that the laptop is missing through reasoning; you have an immediate impression of its absence.”

According to this account, the absence of a laptop is directly perceived, instantaneously and without any conscious effort, as a mismatch of sensory input relative to a perceptual template of a laptop on a table. This seems to contrast with the account presented here in several ways.

First, according to this account, absence is perceived, whereas in the account I defend it is inferred. On closer inspection, this is not in fact a point of disagreement. Perception is widely held to involve, and depend on, inference from noisy sensory data about unknown world states (Friston, 2010; Gershman et al., 2012; von Helmholtz, 1948). Therefore, that absence is inferred does not mean that is cannot also be perceived. Indeed, Gow (2021) proposes that absence is perceived via “intellectual seeming”: a form of inference that results not in beliefs or judgments, but in perceptual states.

The next point of potential disagreement concerns what knowledge is necessary to infer absence. According to the template-mismatch account, any sensory mismatch relative to an expected template immediately results in a perception of, or inference about, absence. In the account defended here, absence can only be inferred when one believes that they would have perceived the missing object if it were present. Consider, for example, returning from a break and finding a waiter occluding some of the table. As in Farennikova’s example, the sensory input is inconsistent with your expectation to find your laptop on the table, but this time you are not inferring that it is absent, because you know that the waiter might be occluding it. Similarly, if you believe the laptop would be difficult to see (for example, if you forgot your glasses inside), you will not infer absence until you check the table more closely. In both cases, inference about absence depends on much more than a comparison to a sensory template: it depends on sophisticated inference based on sensory and metacognitive cues.

In defense of a template-mismatch account, one may argue that the difference between seeing the absence of a laptop in Farennikova’s example and not seeing it in my occluding-waiter or missing-glasses variants is not in post-perceptual inferences, but in the sensory templates against which the sensory input is compared. For example, my sensory template of a laptop on a table may itself become less clear when I know the lighting has changed. Critically, this flexible updating of sensory templates based on changing environmental and internal conditions is a model-based process, one that involves not only modelling of objects and other agents, but of my own perception and attention too.

Finally, in support of the template-mismatch account, Farennikova mentions that many experiences of absence feel instantaneous and lacking in conscious effort, indicating some automaticity of absence processing. However, introspection can be misleading. In perception, inference about absence is significantly slower than inference about presence or stimulus type, even when presenting the decision as a discrimination task between two stimuli (Mazor et al., 2021).^1^

To conclude, a template-mismatch account of inference about absence as the one put forward by Farennikova (2013) either includes implicit self- and world-modelling in the generation of context-sensitive templates, or fails to account for the flexibility with which subjects infer absence in dynamic environments and internal conditions.

### The “Difference” Heuristic

In a computational model proposed by Gold and Shadlen (2001), criterion setting is rendered entirely unnecessary by adopting what I will term here a “difference heuristic”. In this model, when making a binary decision about whether a dim light is on or off, decision centers in the brain can focus on the difference between evoked responses (for example, spiking rates) in two neuron ensembles that are sensitive one to the presence and one to the absence of light. Using this difference heuristic, the decision criterion can always be set at 0: a positive difference between these two quantities indicates that a light is more likely to be present, and a negative difference indicates that a light is more likely to be absent. The difference heuristic can also be used to model reaction times in a sequential evidence accumulation setting. For example, participants may only commit to a response once the summed difference in neural activation between light-sensitive and darkness-sensitive neurons crosses a certain decision boundary (as in some formulations of the popular Drift Diffusion Model; Bogacz et al., 2006; Ratcliff & McKoon, 2008). Critically, the difference heuristic requires no knowledge of the underlying likelihood functions going from world states to activation patterns.

But for this heuristic to be valid, the brain must represent the two candidate world states in a symmetric fashion. To continue with the light detection example, there should be a pair of neuron ensembles *n*_1_ and *n*_2_, such that the distribution of responses of *n*_1_ to the presence of light is indistinguishable from the distribution of responses of *n*_2_ to the absence of light, and vice versa. If *n*_1_ responses (*x*_1_) are stronger on average than *n*_2_ responses (*x*_2_), the difference *x*_1_ − *x*_2_ would tend to be positive, introducing a persistent bias in the agent’s responses and degrading their accuracy as a result. While it is easy to imagine such perfectly symmetrical response profiles in low-dimensional representation spaces, such as rightward versus leftward motion energy in motion discrimination tasks, it is more difficult to see how this heuristic can be practically useful in more ecological settings, where representations are complex and high dimensional. For example, in deciding whether an ambiguous figure is a dog or a cat, is it warranted to assume that cat-sensitive neurons have similar response profiles to cats as do dog-sensitive neurons to dogs, and vice versa?

The limitations of the difference heuristic become much more apparent in a detection setting, where decisions are made about the presence or absence of objects. Sensory neurons commonly encode the presence of features, not their absence. For example, while we expect some neurons (for example, in the ventral visual stream and medial temporal lobe) to show specificity to the representation of a cat, it would make no sense for the brain to also have neurons that respond to the absence of cats. For most people, these hypothetical cat-absence neurons would be constantly firing, together with the neurons that represent the absence of one’s grandma, of zebra stripes, of blue feathers, and of many other objects, agents and features. Even in simpler detection tasks, such as detecting a vertical grating with an arbitrary phase in random visual noise, it is unclear whether there are neurons that respond whenever there is no sign of a vertical orientation, or no sign of a specific spatial frequency. The difference heuristic works when two world states are symmetrically represented, but this is almost never the case in contrasts between presence and absence. This makes the difference heuristic a poor model for most detection decisions, and for decisions about absence specifically.

### Adapting Decision Policy Parameters Based on Task Experience

In psychological experiments, individual decisions about presence and absence are commonly performed as part of a block of similar decisions. This allows subjects to adaptively change their decision policy to maximize accuracy in a model-free way, that is, without any updating of world or self-models.

For example, when accuracy feedback is delivered in a visual search task, missing the presence of a target slows down subsequent ‘target absent’ responses, without an effect on ‘target present’ responses (Chun & Wolfe, 1996). According to the model proposed by the authors, only items that exceed an activation threshold are selected for serial scanning (starting from the item with highest activation and going down), and a ‘target absent’ response is given only once the last selected item is classified as non-target. A lower activation threshold allows more items to be selected, resulting in longer ‘target absent’ responses, with no effect on ‘target presence’ responses. By dynamically updating the threshold based on error trials, subjects can make accurate and efficient inferences about absence without having any internal representation of their own perception or cognition.

Similarly, sequential dependencies in perceptual detection suggest that subjects may update the SDT decision criterion based on previous trials (Dorfman & Biderman, 1971; Kac, 1962), even when no feedback is available (Thomas, 1975). In different contexts, the decision criterion may be gradually adapted to stabilize the proportion of target-present responses at 50% (reflecting a belief that a target should be present in 50% of the trials), or to track changes in the probability of a target to be present (reflecting a belief that target-present trials tend to cluster together; Treisman & Williams, 1984). Relatedly, foragers’ decisions to terminate their search for food in a given area and move to the next one can be modeled as a model-free process, updating a single parameter which tracks the global rate of return (for example, number of berries per minute) in the global environment (Charnov, 1976)—no world- or self-models required.

For these classes of models, the question remains how participants set the values of these decision policy parameters in the very first trial of an experiment, or in cases where only one decision has to be made (Treisman & Williams, 1984; Wolfe, 2021). One possibility is that decision policy parameters are initially given arbitrary values, which slowly converge to their optimal position via parameter adjustment heuristics. An alternative is that initial values are chosen in an informed way, based on prior expectations about perception and attention. In support of the second option, we recently found that subjects make efficient decisions about target absence in the very first trial of a visual search task, before any parameter adjustment can take place (Mazor & Fleming, 2022). Subjects searched for a red dot among blue dots (easy search) or among blue dots and red squares (hard search). The order of trials and item locations were randomized, with the exception that a target was never present in the first four trials. Between-subject comparisons revealed that target-absent responses in trial 1 were fast and unaffected by set size in the easy search, but slow and sensitive to set size in the hard search condition. This result indicates that in addition to adaptive parameter adjustment, decision heuristic parameters are set in alignment with more stable expectations about perception and attention.

## USING INFERENCE ABOUT ABSENCE TO STUDY THE MENTAL SELF-MODEL

In this paper I argue that the mental self-model plays an important role in inference about absence. I provide examples from near-threshold perception, visual search, and long-term memory, for cases where accurate beliefs about one’s own perception and cognition can increase the accuracy, speed, and metacognitive access to the quality of decisions about the absence of objects or memories. This makes inference about absence a unique window into the mental self-model, and critically, one that does not depend on introspective awareness.

If inference about absence draws on knowledge from a mental self-model, behavioural markers of such inferences (such as decision time, accuracy, and subjective confidence) can be used to identify the parameters of the self-model. In other words, these measures can arbitrate between competing mental self-models that subjects may have at the time of performing the task. In the above examples, behaviour was used to identify qualitative properties of the self-model, such as an exaggerated effect of attention on perceptual sensitivity, or no knowledge of the immediate capture of attention by unfamiliar stimuli. This approach can be taken one step further by specifying a model family and identifying model parameters that agree with the observed data.

As an example, consider the effect of attentional manipulations on detection decisions and confidence ratings. As illustrated in Figure 3, the findings of Kellij et al. (2021) are qualitatively consistent with subjects having miscalibrated metacognitive beliefs about the effect of attentional capture on detection performance. Specifically, if subjects overestimate attention effects, they may overcompensate for them by adjusting their decision criterion on different trials, resulting in an inversion of the relative variance of target-present and target-absent SDT properties. Do subjects merely overestimate these effects, or do they have a qualitatively different internal model of their attention (for example, one where attention is modeled in a binary fashion, as being either on or off)? Different metacognitive beliefs about the effects of attention on perception imply different optimal strategies for criterion settings, which can be quantitatively compared against empirical data from detection experiments involving experimental manipulations of attention.

An advantage of this approach is that it does not depend on explicit metacognitive evaluations. Metacognitive knowledge is typically probed in the lab by means of explicit report, for example, by asking subjects to rate their ability or make prospective confidence ratings (Fleming et al., 2016). The examples in this paper demonstrate that some self-knowledge can be accessible only to some subsystems, encapsulated from introspection. Extracting the contents of the mental self-model based on inference about absence may, in some cases, reveal self-knowledge that is not available for explicit report but is used to guide behaviour nonetheless. Importantly, not being available to report does not mean this knowledge is model-free or hard-wired (cf. Carruthers, 2018; Carruthers & Williams, 2022). Metacognitive knowledge about one’s own perception, attention and memory can be model-based and used flexibly in different settings, while still being inaccessible to report, similar to how knowledge of grammar rules in one’s mother tongue can be used to form sentences without being available in the form of declarative knowledge.

This indirect approach can be highly beneficial in the developmental study of babies and infants, who may not be able to provide reliable explicit metacognitive ratings due to limited communication skills or the lack of an explicit theory of mind, but whose implicit mental self-model is growing and changing in interesting ways. For example, in perception, the abilities to represent absences and presences show a different developmental trajectory. Four month old babies show preferential looking for novel presences, but not for novel absences (Coldren & Haaf, 2000), and eight month old babies are surprised when the magical disappearance of objects, but not by their magical appearance (Wynn & Chiang, 1998). In the context of the framework presented here, the acquisition of the ability to actively represent absences may reflect the gradual expansion of different aspects a mental self-model, and the development of the capacity to use this model for counterfactual reasoning.

The development of the self-model can be studied in adults too. Similar to models of the world or of one’s body and motor system, a mental self-model is expected to expand and change in light of new evidence, and these changes will be evident in decisions about absence. For example, in discussing inference about absence in the context of memory, I described a study where participants were led to believe that high usage frequency made words more or less memorable (Strack et al., 2005). These beliefs were later reflected in participants’ tendency to categorize high and low frequency words as ‘old’ or ‘new’. In one experiment, belief induction was obtained without explicitly telling participants which words were more memorable. Instead, Strack and colleagues made use of the fact that high-frequency words are more easily recalled in free-recall paradigms, but low-frequency words are more easily recognized in item recognition paradigms. An additional free-recall/item-recognition task prior to the main recognition memory test induced different beliefs about item memorability in the two experimental groups. These newly acquired beliefs were reflected in participants’ negative recognition judgments, without a need to explicitly probe participants’ explicit metacognitive beliefs about word memorability.

## CONCLUSION

An accurate mental self-model is necessary for transforming the absence of evidence for the presence of objects or memories into beliefs about the absence of objects or memories. Findings from the fields of visual psychophysics and recognition memory suggest that this model is sometimes exaggerated or simplified, and that it develops with age and task experience. Here I suggest to utilize the tight link between inference about absence and the mental self-model to empirically study the structure and contents of this model, without assuming that participants have full access to it at all times.

## Acknowledgments

I thank Stephen M. Fleming and Nadine Dijkstra for valuable feedback and comments.

## Note

^1^ Decisions about the absence of memories are found to be slower in some studies (for example, Hoving et al., 1970; Ratcliff & Murdock, 1976; Sternberg, 1969), but this is not always the case. This may have to do with participants exhaustively scanning the entire memory array before committing to a decision, even after the test item is identified as old (Sternberg, 1969).
